# Risk factors for urinary tract infection in patients with urolithiasis—primary report of a single center cohort

**DOI:** 10.1186/s12894-018-0359-y

**Published:** 2018-05-21

**Authors:** Li Yongzhi, Yan Shi, Liu Jia, Liu Yili, Zhu Xingwang, Gong Xue

**Affiliations:** 1 Department of Urology, Urologic Minimally Invasive Treatment Center in Liaoning Province, The Fourth Affiliated Hospital of China Medical University, No4, Chongshan East Road, Shenyang, 110032 China; 2 Department of Urology, Sheyang Red Cross Hospital, No4, Chongshan East Road, Huanggu District, Shenyang, 110032 China

**Keywords:** Urinary tract infection, Risk factors, Urolithiasis, Urine culture test

## Abstract

**Background:**

Urinary tract infection (UTI) is very common in patients with urolithiasis, which makes the treatment of urolithiasis complicated, even dangerous. The objective of this study was to determine the risk factors for UTI in patients with urolithiasis.

**Methods:**

Eight hundred six patients with urolithiasis were retrospectively evaluated in the fourth affiliated hospital of China Medical University. All patients admitted to the study were divided into either a UTI infection group or a non-infection group. Sex, age, smoking, stone shape, alcohol consumption, position of stones, and presence of obstruction were used as exposure factors for the cross-sectional study.

**Results:**

One hundred seventy-eight patients (22.0%) had UTI. Through a urine culture test, gram-negative bacilli were the most common pathogen, followed by gram-positive bacilli and fungi.

**Conclusions:**

Sex, age, obstruction, stone shape, and multiple sites of stones could be considered the independent factors for UTI in patients with urolithiasis; smoking and drinking had no statistically significant correlation with the condition. Gram-negative bacilli are the most common pathogen in UTI in patients with urolithiasis.

## Background

Urolithiasis is one of the most common urological diseases, the prevalence of which ranges from 2.0 to 20% throughout the world based on the geographic and socioeconomic characteristics of different populations, and > $2 billion is spent on treatment each year [1, 2]. The prevalence of urolithiasis appears to have increased in recent years for both men and women [3, 4].

Urinary tract infection (UTI) is very common in patients with urolithiasis. Persistent infections caused by urease-producing bacteria will form infection stones consisting of monoammonium urate, struvite (magnesium ammonium phosphate), and/or carbonate apatite [5], which makes the treatment of urolithiasis complicated. Complications from urolithiasis, such as asymptomatic bacteriuria, UTI, and sepsis, have been recognized after treatment with extracorporeal shock-wave lithotripsy [6]. Patients with severe or multiple stones might develop postoperative systemic inflammatory response syndrome after a percutaneous nephrolithotomy (PCNL), with a small percent progressing to urosepsis, which could lead to a catastrophic even, such as septic shock [7]. Of all infections of the urogenital tract, pyelonephritis is the most severe and leads to dangerous complications [5].

Few studies have been published on the risk factors for infection in patients with urolithiasis. In their studies, Schwartz [8] and Wong [9] found catheter, pouches, urinary tract obstructions, neurogenic bladder voiding disruptions, medullary sponge kidney, and distal renal tubular acidosis to be the risk factors for UTI and the development of infection stones. Li [10] and Liu [11] found that of the study patients with urolithiasis, females and those with diabetes mellitus were more prone to septic shock after PCNL treatment. In addition to these factors, there might be some others related to stone formation. For example, smoking, alcohol consumption [12], and other patient characteristics might influence UTI in patients with urolithiasis. The primary aim of our retrospective cross-sectional study was to analyze the risk factors for UTI in patients with urolithiasis; therefore, we chose sex, age, smoking, alcohol consumption, position of stones, presence of obstruction, and stone shape (whether staghorn stones) as risk factors.

## Methods

### Patients

In our study, data on all patients with urolithiasis were collected from September 2006 to February 2009 in the Fourth Affiliated Hospital of China Medical University. All the experiments were performed in accordance with the guiding principle of Fourth Affiliated Hospital of China Medical University Human Ethics Committee and were approved by the Human Care Committee of the Fourth Affiliated Hospital of China Medical University. Exclusion criteria were antibiotic usage within the previous 3 d; urinary tract instrumentation; and cardiac, renal, or hepatic failure.

### Methods

Ultrasound, X-ray, CT, and intravenous pyelography were used to diagnose and classify the position of the stones, presence of an obstruction, and stone shape (whether staghorn stones) in the radiology department, and a routine urinalysis and urine culture test were performed to diagnose a UTI. UTI was defined as presenting one of the following signs or symptoms: fever of > 37.8 °C with dysuria, frequent urination, urgent urination, and/or suprapubic pain with growth of > 10^5^ colony-forming units (CFUs)/mL from a properly collected midstream “clean-catch” urine sample [13].

Subjects were defined as alcohol drinkers and/or cigarette smokers if they had regularly consumed any alcoholic beverage one or more times per week or had smoked 10 or more cigarettes per week for at least 6.0 months [14].

All patients included in our study were divided into either a UTI infection group or a non-infection group. Sex, age, smoking, alcohol consumption, position of stones, presence of obstruction, and stone shape (whether staghorn stones) were used as risk factors in our study.

### Statistical analyses

All analyses were performed using SPSS version 15.0 (SPSS Inc., Chicago, IL, USA). All data are reported as the mean ± SD. Univariate analyses were performed using Student’s t-test for parametric variables and the Kruskal–Wallis test for nonparametric variables to detect influencing factors for UTI. The chi-squared test and Fisher’s exact test were used for comparing ratios. A post-hoc statistical analysis was used for comparing 3 subgroups in age group. *P* < 0.05 was considered statistically significant.

## Results

Eight hundred six patients were included in our study of whom 178 (22.0%) had UTI. The general results are provided in Table 1. Ureteral calculi were the most common type of condition, followed by renal calculi, bladder stones, and urethral stones.

**Table 1:** Different locations of urolithiasis

Stone position	Number of cases	%
Renal stone	274	34.00
Ureteral stone	280	34.74
Bladder stone	50	6.20
Urethral stone	11	1.36
Multiple sites	191	23.70
Total	806	100.00

Table 2 shows the results of the urine cultures. Gram-negative bacilli isolates were the most common pathogen, followed by gram-positive bacilli isolates and strains of fungi (93.3 vs 4.5 vs 2.3%, respectively). Among the gram-negative bacilli, *Escherichia coli* was the most common, followed by *Pseudomonas aeruginosa*, *Klebsiella pneumoniae*, *Proteus mirabilis*, and “other” (52.80, 15.16, and 12.35% respectively).

**Table 2:** Bacterial species and ratio in total

Bacterial species	Isolates count	%
Gram-negative bacteria	166	93.25
*Escherichia coli*	94	52.80
*Pseudomonas aeruginosa*	27	15.16
*Klebsiella pneumoniae*	22	12.35
*Proteus mirabilis*	7	3.93
Miscellaneous	16	8.98
Gram-positive bacteria	8	4.49
Fungus	4	2.24

Female patients had a higher rate of infection than male patients (32.0 vs 15.8%, *P* < 0.001) and patients > 60 years old were more prone to be infected, followed by those < 40 years old and 40–60 years old (31.0 vs 23.0 vs 18.3%, respectively; *P* = 0.009). Patients with obstructions were more prone to be infected than those without obstructions (26.1 vs 18.2%; *P* = 0.006). Patients with multiple stones had a higher rate of infection than those with a single stone (41.3 vs 16.0%, *P* = 0.001). Patients who smoked had a higher rate of infection than those who did not smoke (25.8 vs 20.1%, *P* = 0.063), but patients who drank alcohol had a lower rate of infection than those who did not drink alcohol (21.8 vs 22.2%, *P* = 0.906). Patients with staghorn stones had a higher rate of infection than those without staghorn stones (48.4 vs 19.8%, *P* < 0.001). (see Table 3).

**Table 3:** Different risk factors for urinary tract infection

Risk Factors	Number	Without Infection		With Infection		χ^2^	P
		N	%	N	%		
Sex						28.49	<.0001
Male	492	414	84.15	78	15.85		
Female	314	214	68.15	100	31.85		
Age group^a^						9.35	0.0093
<40	252	194	76.98	58	23.02		
40-60	409	334	81.66	75	18.34		
>60	145	100	69.66	45	31.03		
Stone site						54.06	<.0001
Single stone	615	516	83.90	99	16.10		
Multiple sites	191	112	58.64	79	41.36		
Obstruction						7.38	0.0066
Yes	394	291	73.86	103	26.14		
No	412	337	81.80	75	18.20		
Staghorn calculi						28.06	<.0001
Staghorn stones	64	33	51.56	31	48.44		
Non-Staghorn stones	742	595	80.19	147	19.81		
Smoking						3.44	0.0638
Yes	279	207	74.19	72	25.81		
No	527	421	79.89	106	20.11		
Alcohol Drinking						0.016	0.9006
Yes	293	229	78.16	64	21.84		
No	513	399	77.78	114	22.22		

^a^Age group,

<40 versus 40-60,χ^2^ = 3.08, *P* = 0.0793;

<40 versus >60, χ^2^ = 2.12, *P* = 0.1451;

40-60 versus >60, χ^2^ = 10.17, *P* = 0.0014;

Besides, we did separate analysis of patients with staghorn stone in comparison with non-staghorn stone, and we found the results were almost the same. However, there are two differences.

The first difference is that age does not have a statistically significant relationship to UTI in staghorn stone group (*P* = 0.2150), however, age does have a statistically significant relationship to UTI in non-staghorn stone group (*P* = 0.0215). (see Tables 4 and 5). We guess the reason may be that the number of patients with staghorn stone is a little small.

**Table 4:** Different risk factors for urinary tract infection in Staghorn stones

Risk Factors	Number	Without Infection		With Infection		χ^2^	P
		N	%	N	%		
Sex						37.16	<.0001
Male	39	32	82.05	7	17.95		
Female	25	1	4.00	24	96.00		
Age group						3.07	0.2150
<40	20	9	45.00	11	55.00		
40-60	32	20	62.50	12	37.50		
>60	12	4	33.33	8	66.67		
Smoking						0.03	0.8563
Yes	22	11	50.00	11	50.00		
No	42	22	52.38	20	47.62		
Alcohol Drinking						0.35	0.5521
Yes	23	13	56.52	10	43.48		
No	41	20	48.78	21	51.22

**Table 5:** Different risk factors for urinary tract infection in Non-Staghorn stones

Risk Factors	Number	Without Infection		With Infection		χ^2^	P
		N	%	N	%		
Sex						12.53	0.0004
Male	453	382	84.33	71	15.67		
Female	289	213	73.70	76	26.30		
Age group						7.68	0.0215
<40	232	185	79.74	47	20.26		
40-60	377	314	83.29	63	16.71		
>60	133	96	72.18	37	27.82		
Smoking						3.81	0.0509
Yes	257	196	76.26	61	23.74		
No	485	399	82.27	86	17.73		
Alcohol Drinking						0.01	0.9223
Yes	270	216	80.00	54	20.00		
No	472	379	80.30	93	19.70		

The second difference is that the first three gram-negative bacteria in staghorn stone are *Escherichia coli*, *Pseudomonas aeruginosa and Klebsiella pneumoniae*. However, the first three gram-negative bacteria in non-staghorn stone are *Escherichia coli, Klebsiella pneumoniae and Pseudomonas aeruginosa.* (see Tables 6 and 7).

**Table 6:** Bacterial species and ratio in Staghorn stones

Bacterial species	Isolates count	%
Gram-negative bacteria	31	100.00
*Escherichia coli*	12	38.70
*Pseudomonas aeruginosa*	11	35.48
*Klebsiella pneumoniae*	5	16.12
Miscellaneous	3	9.67

**Table 7:** Bacterial species and ratio in Non-Staghorn stones

Bacterial species	Isolates count	%
Gram-negative bacteria	135	91.83
*Escherichia coli*	82	55.78
*Klebsiella pneumoniae*	17	11.56
*Pseudomonas aeruginosa*	16	10.88
*Proteus mirabilis*	7	4.76
Miscellaneous	13	8.84
Gram-positive bacteria	8	5.44
Fungus	4	2.72

Through the results of the chi-squared tests, sex, age, obstruction, multiple stones, and stone shape each had a statistically significant relationship to UTI (all *P* < 0.05); however, this was not true of smoking and alcohol consumption (all *P* > 0.05).

## Discussion

In our retrospective study, the independent effects of risk factors on the development of UTI were investigated. Sex, age, obstructions, stone shape, and multiple sites of stones were found to be the independent risk factors for UTI in patients with urolithiasis, which might be helpful in their treatment.

Previous reports showed that females had a higher rate of infection stones than males [15, 16]. In Li and Liu’s study [10, 11], females with urolithiasis were found to be more prone to septic shock after PCNL treatment. These results were comparable to ours. The reason might be that women have a shorter urethra, which predisposes them to ascending infections. Nearly 10% of women experience infections of the urinary tract within 1.0 year of PCNL treatment, including cystitis and pyelonephritis [17], and as many as 26% of UTIs recur within 6.0 months [18].

In our study, the patients < 40 years old and > 60 years old were more prone to infections than those from 40 to 60 years old. Thomas [16] found that infection stones were most common in each sex at ages from 60 to 69 years compared with that in other age groups, which was nearly the same as indicated from our results. Daudon et al. [15] reported that struvite was especially low in ages 40 to 49 years, but the frequency peak of ammonium urate stones was observed in ages 0 to 9.0 years. Thereafter, the proportion declined rapidly. In our study, patients between the ages of 40 and 60 years showed a low rate of infection compared with that of the other two groups.

It has been proved that urinary tract obstruction is a risk factor for UTI and the development of infection stones urine cannot pass smoothly. In addition, the inflamed narrowing of the ureter or injuries made by stones when moving down the ureter could easily cause infection. In our study, obstructions were confirmed by CT scan and intravenous pyelography, and 26.14% of patients with obstructions were prone infection compared with 18.20% of patients without obstructions, the results of which were the same as those in the previous study [5]. In addition, those with multiple stones are more likely to be infected than those with a single stone, which could be because multiple stones have more of a chance to cause an obstruction, which could easily cause urinary retention after which the chance of UTI increases significantly.

Staghorn calculi are branched stones that occupy a large portion of the collecting system. Typically, they fill the renal pelvis and branch into several or all of the calices. However, there is no consensus regarding the precise definition of staghorn calculus, such as the number of involved calices required to qualify for a staghorn designation. In our study, the term staghorn stone refer to any branched stone occupying more than one portion of the collecting system, ie renal pelvis with one or more caliceal extensions [19]. A staghorn calculus has traditionally been synonymous with infection stones. Typically, UTI with urease-producing bacteria promote the crystallization and formation of branching stones that encompass the renal pelvis and calyces [20]. It was reported that in 59–68% of cases, the majority of infectious constituents were staghorn calculi [21], which suggests that patients with staghorn calculi are more easily infected. In our study, patients with staghorn calculi were also more likely to be infected, which confirmed the results of previous reports.

There was no statistical significant correlation between smoking and/or alcohol consumption and infection, which indicates that smoking and alcohol consumption could not be considered as independent risk factors for UTI in patients with urolithiasis; however, recently, cigarette smoking has been identified as an important risk factor for the development and progression of urolithiasis [12]. Decreasing urinary flow [22] and increasing serum cadmium [23] might be two reasons for urolithiasis that are associated with smoking and alcohol consumption. In healthy subjects, smoking has been found to significantly increase the antidiuretic plasma arginine vasopressin, resulting in a decrease in urinary flow and possibly promoting the development of calcium urolithiasis [12]. Hamano et al. [24] reported that cigarette smokers have a 4.29-fold risk of developing calcium urolithiasis; however, they did not report any correlation between smoking and UTI.

Typically, gram-negative bacteria are the most common pathogen of UTI, among which, *E. coli* has a high frequency rate [25]. In epidemiology and antimicrobial susceptibility profiles of gram-negative bacteria causing UTIs in the Asia-Pacific region, Lu et al. [26] reported that *E. coli*, *K. pneumoniae*, and *P. aeruginosa* were the three most common species of pathogens found in UTIs. In our study, gram-negative-bacteria were the most common, followed by gram-positive bacteria and fungus. Among the gram-negative-bacteria, *E. coli* was the most common pathogen following by *P. aeruginosa*, *K .pneumoniae* and *P. mirabilis*.

Our study had a number of limitations. First, we lacked the data on stone composition, which could be useful for analysis of infection stones. Second, we lacked the details of antimicrobial susceptibility testing. Thus, the relationship between stone composition and bacterial colonization received our attention and might be the subject of our next study.

## Conclusions

Sex, age, obstruction, multiple sites of stones, and stone shape (whether staghorn stones) could be considered as independent factors for UTI in patients with urolithiasis. Gram-negative bacilli are the most common bacteria found in UTIs in patients with urolithiasis.

## Abbreviations

CFUs: Colony-Forming Units
PCNL: Percutaneous Nephrolithotomy
SD: Standard Deviation
UTI: Urinary Tract Infection
